# Supplementary material for: Assessing the ecological impacts of transportation infrastructure development: A reconnaissance study of the Standard Gauge Railway in Kenya
Source: PLoS One. 2021 Jan 29;16(1):e0246248. doi: 10.1371/journal.pone.0246248 (PMC7845991; doi:10.1371/journal.pone.0246248)
Supplement: S1 Appendix — (DOCX) [file pone.0246248.s001.docx]

| **Category** | **Code** | **Description** | **Count** | **% Codes** |
| --- | --- | --- | --- | --- |
| What are the ecological impacts of SGR? | Ecosystem Degradation | These are activities resulting in the loss of ecosystem integrity through disturbance of resident species, contamination with pollutants including chemicals, noise and dust, alteration of natural processes such as hydrological dynamics (water flow) fire regimes, and introduction of exotic species and alteration of species interactions | 22 | 55.00% |
| What are the ecological impacts of SGR? | Ecosystem Fragmentation | Activities resulting in the loss of ecosystem integrity through the creation of barriers to movement of terrestrial animals, disruption of normal animal behaviour and activities, destruction of part of a habitat, leaving other areas intact, direct mortality of animals attempting to cross the highway corridor that result from collisions with motor vehicles, resultant negative interactions between wildlife and people such as HWC | 11 | 27.50% |
| What are the ecological impacts of SGR? | Ecosystem Destruction | Activities resulting in the elimination of certain habitat types and their replacement with non-natural uses or with specialized semi-natural habitats. These may include conversion" to a transportation land use or "right-of-way". Although natural vegetation may be preserved within the right-of-way, the original natural characteristics of the land are eliminated within the paved area and adjacent roadside. The clearing of vegetation (trees, shrubs, grasses) and accompanying levelling operations (that destroy the original topography and soil profile) are the principal changes. In some cases, the natural vegetation may be replanted while in others different species are planted and the habitat values modified. In wetland environments, road construction may require filling and draining operations that destroy wetland habitats. In aquatic environments, flow alteration (via damming or channelization) may eliminate habitat. Dredging, filling, and draining required by road construction also destroy aquatic habitat. | 7 | 17.50% |

**List of codes and frequencies**
