# Supplementary material for: Assessing the ecological impacts of transportation infrastructure development: A reconnaissance study of the Standard Gauge Railway in Kenya
Source: PLoS One. 2021 Jan 29;16(1):e0246248. doi: 10.1371/journal.pone.0246248 (PMC7845991; doi:10.1371/journal.pone.0246248)
Supplement: S2 Appendix — (DOCX) [file pone.0246248.s002.docx]

| **Category** | **Code** | **Case** | **Text** | **Coder** | **Date** | **% Words** |
| --- | --- | --- | --- | --- | --- | --- |
| What are the ecological impacts of SGR? | Ecosystem Degradation | CFA Mombasa | Currently, there is a lot of sedimentation coming down from the road and is thought to be affecting mangroves especially in terms of seed development. This was observed by the CFA during the construction phase when they harvested fewer seeds and speculated that the sediment was affecting seed production. Sedimentation is also thought to affect self-germination of mangroves. The stream in the area observed has also been blocked across the road at the estuary and has been reduced in size. | Admin | 26/04/2019 | 18.00% |
| What are the ecological impacts of SGR? | Ecosystem Degradation | Meeting with Community members at Tuala | The team visited a borrow pit left idle by the SGR contractor and which has filled up with water, causing a hazard to the local community and their livestock. The water was observed to be orange in colour due to a chemical that the contractor uses in blasting the rocks. The informants also stated that the water has been found to be unfit for consumption and is instead used only to water the gardens and livestock. | Admin | 26/04/2019 | 16.30% |
| What are the ecological impacts of SGR? | Ecosystem Destruction | Mramaroi Farm | Mr. Kituku stated that he has observed a number of changes in land use in the area, mainly sub-division of land due to population pressure and development of the area through the railway and road network. | Admin | 26/04/2019 | 14.50% |
| What are the ecological impacts of SGR? | Ecosystem Degradation | Helsinki University Research Centre | There has also been an increase in forest fires in the area. Invasive species are also a problem in the area with Eucalyptus becoming invasive on the hilltops and Acacia mearnsii is killing indigenous species. | Admin | 26/04/2019 | 13.70% |
| What are the ecological impacts of SGR? | Ecosystem Degradation | Meeting with community members in Oloosirkon area | The underpasses have been flooding when it rains and rivers are also impassable because the culverts constructed do not work properly. River Embesh has dried up completely because it was filled with silt from the construction. Other water sources such as R. Mbagathi have also been blocked. | Admin | 26/04/2019 | 12.70% |
| What are the ecological impacts of SGR? | Ecosystem Degradation | Narok County | Clearing of vegetation, the area and directing water to 255 underpasses has led to gulley erosion affecting soil cover and leading to siltation of L. Magadi. 33% of the lake is currently under siltation and is expected to cost over Ksh 100 million per year to scoop out. The siltation is also affecting quality of the mineral Trona which is very valuable globally. The siltation has covered area where they extract the purest material. Sand extraction is also a major problem. Siltation from Magadi will also affect areas such as the Loita forest canopy among others where the wildlife migrates through. | Admin | 26/04/2019 | 10.90% |
| What are the ecological impacts of SGR? | Ecosystem Degradation | Mariakani Station | There were a number of issues identified when construction was going on, including dust pollution. The entire construction took one year since the stations were built concurrently. The road to Kinango past the Mariakani station was impassable during rainy season but has been tarmacked by the government. The main environmental impact currently observed by the staff is noise pollution when the trains are passing. Complaints have been made informally but nothing formal recorded. | Admin | 26/04/2019 | 10.30% |
| What are the ecological impacts of SGR? | Ecosystem Degradation | Meeting with community members in Oloosirkon area | The borrow pits created have also been left with gaping holes which animals and people can get stuck in. Bridges in the area have also been destroyed during the construction and have not been repaired. | Admin | 26/04/2019 | 9.50% |
| What are the ecological impacts of SGR? | Ecosystem Degradation | KEFRI | SGR also passes through the Kibwezi area exacerbating already observed impacts on the river. Previously observed impacts include drying up of Umani springs in Chyulu Hills which supplies water to Kibwezi and as far as Makindu. Impacts on water were also observed during the construction of the SGR and Kitui Road. | Admin | 26/04/2019 | 8.80% |
| What are the ecological impacts of SGR? | Ecosystem Degradation | Meeting with Community members at Tuala | Expected impacts of the SGR include population growth and possible impacts of flood waters. Pupils have also had injuries and had to miss school. Dust pollution is also a challenge and impacts include infections from dust, coughs and chest pain. | Admin | 26/04/2019 | 8.60% |
| What are the ecological impacts of SGR? | Ecosystem Destruction | CFA Mombasa | The three main ones the team encountered were Ceriops tagal, Rhizopora micronata and Racesmosa sp., which is locally threatened. The mangroves grow together with supporting vegetation which previous studies have shown indicate mangrove establishment. | Admin | 26/04/2019 | 7.60% |
| What are the ecological impacts of SGR? | Ecosystem Degradation | Mombasa County | Mombasa is low-lying and gets affected by floods. Excavations for roads have also destroyed chunks of land and plant species plus led to unintended collection of water. This led to fears of increased incidences of Chikungunya disease with slums being especially vulnerable to infections. The county government recently had to do fogging to kill mosquito breeding grounds. | Admin | 26/04/2019 | 6.80% |
| What are the ecological impacts of SGR? | Ecosystem Degradation | Meeting with Community members at Tuala | The blasting has also affected the students, some of whom are scared of the blasting. The public health officer had condemned the school because of the condition of the buildings. | Admin | 26/04/2019 | 6.40% |
| What are the ecological impacts of SGR? | Ecosystem Degradation | Meeting with community members in Oloosirkon area | Blasting for materials for construction is causing tremors in the area which have caused buildings to crack, for example at Oloosirkon primary school | Admin | 26/04/2019 | 6.20% |
| What are the ecological impacts of SGR? | Ecosystem Degradation | KWS Tsavo Conservation Area Office | Invasive species also a problem in both parks because of livestock incursion e.g. in Bachuma East. Soil degradation is a major issue in southern part of Tsavo East. The invasive cactus Opuntia is a major problem in the area. Prosopis juliflora (known locally as Mathenge) is also affecting dry river beds in the area and is prevalent along new highway from Voi to Taveta | Admin | 26/04/2019 | 6.20% |
| What are the ecological impacts of SGR? | Ecosystem Degradation | Meeting with Community members at Tuala | The team were also able to observe impacts of the blasting on structures in the area including cracking walls in local houses and at Oloosirkon primary school. | Admin | 26/04/2019 | 5.80% |
| What are the ecological impacts of SGR? | Ecosystem Degradation | WRA | The main impacts of the SGR identified by the team was encroachment on riparian lands in areas where it passes through | Admin | 26/04/2019 | 5.00% |
| What are the ecological impacts of SGR? | Ecosystem Fragmentation | Meeting with community members in Oloosirkon area | Loss of livestock from hyenas has increased since hyenas use underpasses constructed for the SGR to access livestock. | Admin | 26/04/2019 | 4.90% |
| What are the ecological impacts of SGR? | Ecosystem Fragmentation | Helsinki University Research Centre | Human-human conflict is becoming common between upstream and downstream water users. | Admin | 26/04/2019 | 4.70% |
| What are the ecological impacts of SGR? | Ecosystem Degradation | Meeting with community members in Oloosirkon area | Dust pollution and noise pollution from the construction, which sometimes went on for 24 hours a day | Admin | 26/04/2019 | 4.60% |
| What are the ecological impacts of SGR? | Ecosystem Destruction | Taita Taveta County Offices | Land value has gone up due to SGR so led to a lot of development and also migration. Agriculture has also grown because markets in Msa and Nairobi are easily available. | Admin | 26/04/2019 | 4.40% |
| What are the ecological impacts of SGR? | Ecosystem Destruction | Wildlife Works | Water shortages have become more severe. For example, in 2018, seasonal rivers in Taita Hills dried up for the first time. Voi river also runs for only one week a year now. Land subdivision is also a major challenge and subsistence agriculture in the area is becoming unsustainable due to small land sizes | Admin | 26/04/2019 | 4.10% |
| What are the ecological impacts of SGR? | Ecosystem Degradation | Narok County | Water sources have been depleted because of clearing of vegetation and also unsustainable use. For example a borehole at a certain area was drilled and started bringing up steam after nine months of use. | Admin | 26/04/2019 | 3.70% |
| What are the ecological impacts of SGR? | Ecosystem Fragmentation | Narok County | The county is currently carrying out spatial planning. Real estate development is picking up in Suswa. Land sales are high especially around the proposed station in Suswa, mainly in anticipation of the dry port. | Admin | 26/04/2019 | 3.70% |
| What are the ecological impacts of SGR? | Ecosystem Fragmentation | Taita Taveta County Offices | A few reports of wildlife and livestock being hit by SGR. Complaints about human-wildlife (especially elephants and lions) conflict have increased in the recent past. | Admin | 26/04/2019 | 3.70% |
| What are the ecological impacts of SGR? | Ecosystem Degradation | KWS Tsavo Conservation Area Office | The main illegal activity at underpasses is passing livestock through. Previously, protected areas had buffer zones where communities had access but didn't use, but the current population pressure is leading to people entering those buffer ones. | Admin | 26/04/2019 | 3.50% |
| What are the ecological impacts of SGR? | Ecosystem Degradation | KEFRI | There has also been observation of an unrecognised invasive plant species that springs up where degradation has taken place | Admin | 26/04/2019 | 3.30% |
| What are the ecological impacts of SGR? | Ecosystem Degradation | Mombasa County | The main water sources are seasonal rivers e.g. Jitoni and Tsalu rivers. There is a challenge of flooding during rainy season and no water during dry seaso | Admin | 26/04/2019 | 3.30% |
| What are the ecological impacts of SGR? | Ecosystem Degradation | Narok County | Regeneration of an alien species which is common in Kajiado has also occurred. This an invasive weed which invades grazing areas especially towards L Magadi. It's known locally as Oltiemelentei | Admin | 26/04/2019 | 3.30% |
| What are the ecological impacts of SGR? | Ecosystem Fragmentation | Wildlife Works | There has been an observed increase in human-wildlife conflict which may not necessarily be attributed to the SGR since the Mombasa-Nairobi highway has also been seen to affect wildlife movements, for example with animals congregating along the highway | Admin | 26/04/2019 | 3.10% |
| What are the ecological impacts of SGR? | Ecosystem Destruction | KEFRI | Many trees belonging to KEFRI were cleared in the area and KEFRI have so far not been compensated | Admin | 26/04/2019 | 3.10% |
| What are the ecological impacts of SGR? | Ecosystem Destruction | Voi SGR station | The team noted that structures blocking the underpasses are illegal, mainly set up by pastoralists while they are in the area. | Admin | 26/04/2019 | 3.10% |
| What are the ecological impacts of SGR? | Ecosystem Fragmentation | Wildlife Works | Currently, WW have observed some changes in wildlife behaviour caused by the SGR and Mombasa Nairobi highway. For example, it is hard to get elephants across the highway and then across SGR crossing points. | Admin | 26/04/2019 | 2.60% |
| What are the ecological impacts of SGR? | Ecosystem Degradation | Narok County | Negative ecological impacts are also being felt e.g. flooding. Water routes and pathways have also changed. | Admin | 26/04/2019 | 1.80% |
| What are the ecological impacts of SGR? | Ecosystem Fragmentation | KWS Tsavo Conservation Area Office | Old railway line had rail kills and the highway also has a lot of road kills | Admin | 26/04/2019 | 1.50% |
| What are the ecological impacts of SGR? | Ecosystem Fragmentation | KWS Tsavo Conservation Area Office | Construction of SGR electric fence being completed but in the meantime, some wildlife have been killed | Admin | 26/04/2019 | 1.50% |
| What are the ecological impacts of SGR? | Ecosystem Fragmentation | KWS Tsavo Conservation Area Office | HW conflict is on the rise and demonstrations are the order of the day | Admin | 26/04/2019 | 1.30% |
| What are the ecological impacts of SGR? | Ecosystem Fragmentation | Wildlife Works | A lot of underpasses have also been blocked by human settlements | Admin | 26/04/2019 | 0.90% |
| What are the ecological impacts of SGR? | Ecosystem Destruction | KWS Tsavo Conservation Area Office | Also proliferation of illegal settlements especially in those corridors | Admin | 26/04/2019 | 0.90% |
| What are the ecological impacts of SGR? | Ecosystem Fragmentation | KWS Tsavo Conservation Area Office | Animals having challenges in using the crossings | Admin | 26/04/2019 | 0.70% |
